# Role of *Campylobacter jejuni* Infection in the Pathogenesis of Guillain-Barré Syndrome: An Update

**DOI:** 10.1155/2013/852195

**Published:** 2013-08-13

**Authors:** Kishan Kumar Nyati, Roopanshi Nyati

**Affiliations:** ^1^Immune Regulation Laboratory, World Premier International-Immunology Frontier Research Center (WPI-IFReC), Osaka University, Osaka 865-0871, Japan; ^2^Department of Microbiology, Sanjay Gandhi Postgraduate Institute of Medical Sciences, Lucknow 226 014, India

## Abstract

Our current knowledge on *Campylobacter jejuni* infections in humans has progressively increased over the past few decades. Infection with *C. jejuni* is the most common cause of bacterial gastroenteritis, sometimes surpassing other infections due to *Salmonella*, *Shigella*, and *Escherichia coli.* Most infections are acquired due to consumption of raw or undercooked poultry, unpasteurized milk, and contaminated water. After developing the diagnostic methods to detect *C. jejuni*, the possibility to identify the association of its infection with new diseases has been increased. After the successful isolation of *C. jejuni*, reports have been published citing the occurrence of GBS following *C. jejuni* infection. Thus, *C. jejuni* is now considered as a major triggering agent of GBS. Molecular mimicry between sialylated lipooligosaccharide structures on the cell envelope of these bacteria and ganglioside epitopes on the human nerves that generates cross-reactive immune response results in autoimmune-driven nerve damage. Though *C. jejuni* is associated with several pathologic forms of GBS, axonal subtypes following *C. jejuni* infection may be more severe. Ample amount of existing data covers a large spectrum of GBS; however, the studies on *C. jejuni*-associated GBS are still inconclusive. Therefore, this review provides an update on the *C. jejuni* infections engaged in the pathogenesis of GBS.

## 1. Introduction

Guillain-Barré syndrome (GBS) is an immune-mediated demyelinating polyneuropathy of peripheral nervous system (PNS) characterized by acute or subacute symmetrical ascending motor weakness, areflexia, and mild-to-moderate sensory abnormalities [1]. GBS has now become the most common cause of acute flaccid paralysis with an annual incidence of 0.6–4 cases per 100,000 populations after declining the number of polio cases worldwide [2, 3]. Moreover, about two-thirds of GBS patients usually report antecedent infections in which *Campylobacter jejuni*, Cytomegalovirus, Epstein-Barr virus, and *Mycoplasma pneumoniae* are recognised as triggering agents [4]. Among numerous microbial infections, only *C. jejuni* which is a leading cause of gastroenteritis worldwide [5, 6] is firmly established as a causative agent of GBS [7, 8]. Almost 25%–40% of GBS patients worldwide suffer from *C. jejuni *infection 1–3 weeks prior to the illness [9]. Till date, serology and stool culture have been used in various studies for the detection of antecedent *Campylobacter *infections in GBS patients. Culture [10] and serological [9, 11] studies have proved that *C. jejuni* cause infections in GBS patients. However, only 1 in 1000 patients, who are exposed to *Campylobacter *infection, develops GBS [12]. The isolation rate of *C. jejuni* from stool culture of GBS patients' ranges from 8% to 50% [10] and seropositivity ranges from 24% to 76% [9, 11, 13].

Molecular mimicry and a cross-reactive immune response play a crucial role in the pathogenesis of GBS, at least in those cases with a preceding *C. jejuni* infection and with antibodies to gangliosides [14]. Earlier, GBS was thought to be a single clinical entity which later on classified into different clinical and electrophysiological subtypes: acute inflammatory demyelinating polyneuropathy (AIDP), acute motor axonal neuropathy (AMAN), and a severe AMAN form termed acute motor sensory axonal neuropathy (AMSAN) [15]. In the western countries, AIDP is the most common form of GBS [16] while axonal forms such as AMAN and AMSAN are more frequently reported from southeastern Asian countries such as China, Japan, and India [5, 17–19]. The type of preceding infection and patient-related host factors help to determine the form and severity of the disease. AMAN is mainly characterized by pure motor involvement, frequent antecedent infection by *C. jejuni*, associated with anti-GM1 or anti-GD1a immunoglobulin (Ig) G antibodies, and the electrophysiological features of axonal degeneration and reversible conduction block. Various electrodiagnostic and pathologic studies have shown that *C. jejuni* infection is significantly associated with primary axonal dysfunction [2, 15, 20]; however, several reports are also available suggesting *C. jejuni* infection in the demyelinating subtype [13, 21, 22]. Furthermore, it is also believed that GBS following *C. jejuni* infection may be more severe, for instance, having fulminating disease with quadriplegia and requiring ventilator support within 24–48 h of onset [23–25]. Despite the extensive research on *C. jejuni-*associated GBS, the pathogenesis of the disease after *C. jejuni* infection has been incompletely understood possibly due to the lack of nerve biopsies from patients and suitable animal models. Thus, with a focus on clinical, epidemiological, pathogenetic, immunobiology, and laboratory aspects of the most important human pathogen, *C. jejuni*, this review intends to summarize our current knowledge on the role of its infection in the development of GBS by highlighting our recent findings and selected publications in the field of microbial pathogenesis associated with the disease.

## 2. Epidemiology and Distribution of *C. jejuni *and GBS

**Figure 1:**
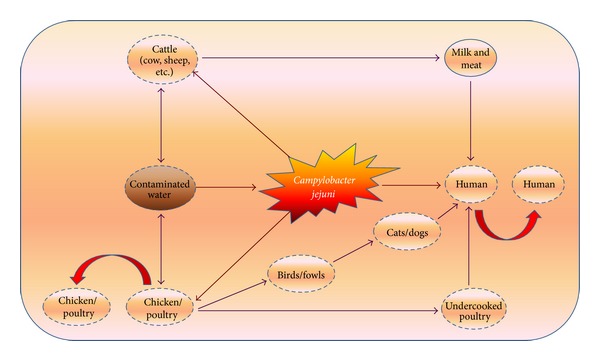
Sources and transmission of* Campylobacter jejuni.* Chicken is a natural reservoir of the *C. jejuni *where it colonizes in the mucosal layer of the gastrointestinal tract and can transfer between chickens through the faecal-oral route. *C. jejuni* can contaminate water and probably form an association with protozoans. Humans who encounter contaminated water, consume undercooked poultry, and unpasteurized milk get infected. The bacterium resides in the epithelial layer of the human gastrointestinal route and causes mainly inflammation and diarrhea. Sometimes antibodies produced against the bacterium mimic with the host nerve gangliosides resulting in demyelination and axonal degeneration of peripheral nerves that causes Guillain-Barré syndrome.

After the culturing of diarrheal stool sample began for enteric pathogens, Campylobacter was identified 2–7 times higher than *Salmonella *or *Shigella spp. *[26]. In the United States, 2.4 million cases of *C. jejuni* including other species were found every year suggesting that it is a more common enteric pathogen [27]. The route of transmission of pathogen is most probably via the fecally contaminated meat surface. There are several other sources including pets and other animals, untreated water and milk, and sewage contamination (Figure 1). In tropical developing countries, *C. jejuni *infections are hyperendemic among young children, especially those aged <5 years. Asymptomatic infections occur commonly in both children and adults, whereas, in developed nations, asymptomatic *C. jejuni *infections are uncommon. On the other hand, in developed nations, outbreaks of infection are unusual and illness lacks the marked seasonal nature observed in industrialized countries.

The incidence of GBS ranges from 0.6 to 4 cases per 100,000 populations every year [2, 3]. Males are more frequently affected than females (1.25 : 1). It occurs in all age groups but the incidence appears to increase with age. Some studies have suggested a possible bimodal distribution of cases with peaks in young adults and elderly [16]. In developed countries, AIDP appears to affect an older population, while in northern China AMAN affects primarily children and young adults [28]. No consistent geographical variations have been reported and most studies have failed to identify the seasonal variation in GBS. However, summer time peaks do occur in China and perhaps in Mexico, Spain, and Korea [13, 28–30]. Several epidemiological studies now firmly established *C. jejuni* as a triggering agent of GBS. Kuroki et al. [31] isolated *C. jejuni* from 30% of GBS patients, whereas Rees et al. had a isolation rate of 8% [32]. In a similar study, *Campylobacter* was recovered from 4 (44.9%) of 9 GBS patients with diarrhea [33]. In a prospective study carried out from our centre, *C. jejuni* and *C. upsaliensis* were detected in patients having AIDP and AMAN type, respectively [34]. Recently, we identified 2.5% and 22.5% of GBS cases with *C. jejuni* infections by culture and PCR, respectively [35]. Overall, the isolation rate of *C. jejuni* from the stool of GBS patients ranges from 8% to 50% [36]. This leads to epidemiological research and consequently to the realization that *C. jejuni *has now emerged as a significant health problem with or without GBS cases throughout the world.

## 3. Association of *C. jejuni* with GBS and Its Subtypes

*Campylobacter jejuni* is considered to be a commensal organism of chicken gut [37] and the leading causative agent of gastroenteritis in humans worldwide [38]. Although the experimental infection of chickens with *C. jejuni* can lead to diarrhea, chickens sometimes can develop severe paralysis resembling neuropathy [22, 39]. The link between *C. jejuni* infection and the development of GBS was first reported in 1982 in a 45-year-old man who developed GBS with irreversible neurological damage two weeks after *C. jejuni*-associated gastroenteritis [40]. Shortly thereafter numerous reports described patients who developed GBS following *C. jejuni* infections [41–44]. In the earlier studies, we have also shown that *C. jejuni *was the most common preceding infection among GBS patients by both serology (26.0%) [45] and lymphocyte transformation test (77.5%) [35]. The seropositivity of *C. jejuni* infection in GBS patients ranges from 24% to 76% among which the highest has been reported from China in AMAN and 42% in AIDP patients [13] suggesting that *C. jejuni *infection elicits AMAN more frequently than AIDP, but a considerable number of AIDP cases also occur after *C. jejuni *infection. In a large study conducted in North America and Europe involving 229 GBS patients, 52 (22.7%) patients had positive serology for *C. jejuni*, and 56% of them showed demyelinating neurophysiology [46]. A study in Japan investigating 86 GBS patients showed that of the 20 (23.3%) *C. jejuni*-positive patients, 70% had AMAN and 15% had AIDP [20]. These results raise the possibility that *C. jejuni *infection can elicit both axonal and demyelinating subtypes of the disease.

## 4. Risk of GBS following *C. jejuni* Infection

GBS that occurs after *C. jejuni *infection is usually more severe related with extensive axonal injury. In addition, a greater likelihood for the need of mechanical ventilation and an increased risk of irreversible neurological damage are also associated. The risk of developing GBS is increased after infection with certain *C. jejuni *serotypes. In the United States, Penner type O:19 is most commonly associated with GBS [47]; in South Africa, Penner type O:41 is frequently reported with GBS. In contrast, the severity of *C. jejuni *infection is not associated with an increased risk of the development of GBS. Although *C. jejuni *infections are common in general population, the risk of developing GBS after *C. jejuni* infection is actually quite low (1 in 1000 patients develops GBS following *C. jejuni* infection) [48] suggesting that the host genetic factors are involved in developing the disease.

## 5. Severity of GBS after *C. jejuni* Infection

Infections due to *C. jejuni *are not normally associated with high rates of mortality in developed countries. Mortality remains around 8% with about 20% of patients with GBS remaining disabled within the first year after onset [49]. It is also believed that GBS following *C. jejuni* infection may be more severe than that caused by other infectious agents. The severity in GBS may sometimes be turned into fulminating disease with quadriplegia requiring ventilator support within 1-2 days of disease onset [25]. Slower recovery, being unable to walk unassisted up to 6 months or 1 year after the onset of disease [32, 50], and severe residual disability and axonal degeneration [13, 32] are the symptoms of such types of GBS cases. However, large prospective studies are needed to confirm these issues.

## 6. Detection Methods of *C. jejuni* Infection in GBS

### 6.1. Culture

Isolation of *C. jejuni *from stool culture is the standard for the detection of infection, but culture would underestimate the frequency of *C. jejuni *infection because the time between the infection and onset of GBS often exceeds the duration of excretion of viable *C. jejuni *in stools [36]. However, culture is still being used to detect *C. jejuni *infection in GBS patients. Sensitivity of culture has been found to be very low in various studies [4, 31, 32, 35]. Culture provides a definitive evidence of *C. jejuni* infection in GBS patients, but due to short median excretion period (16 days) of *C. jejuni *in stool and 1–3 weeks lag time between episode of diarrhea and development of GBS, it miscalculates the infection in these patients. Culture is insensitive to the detection of bacteria in patients treated with antibiotics, or in patients having mild/subclinical infection or in patients with late reactive complications such as arthritis and GBS or long-lasting intestinal distress [51]. Delayed hospital admission and intake of antibiotics by the patient may also account for low culture positivity. Furthermore, culture of stool samples for *Campylobacter *has been done only in GBS cases with severe diarrhea, thereby missing cases in which the infection may be mild or subclinical [52]. 

### 6.2. Enzyme-Linked Immunosorbent Assay (ELISA)

Serologic studies are more sensitive but less specific than culture-based methods. There are no standards for serologic testing for *C. jejuni *infection with regard to antigens used or cutoff values for the positivity, and the sensitivity and specificity of serologic assays vary considerably among laboratories [53]. Serology is mainly used to detect the presence of antibodies against *C. jejuni* infection in patient's serum. Serum IgA and IgM levels rise in response to infection and remain elevated for 3-4 weeks before declining to baseline levels [54], but serum IgA levels rise during the first few weeks of infection and then fall rapidly [54, 55]. Several drawbacks are associated with serology too: there is no consensus on the choice of antigens; most often a crude antigenic extract and single serum sample are used yielding low specificity, especially in endemic and hyperendemic countries due to high titres of antibodies in the resident population [35, 52]. Furthermore, testing of paired sera and demonstration of significant increase and decrease in antibody titer may be required which is difficult and depends on the time of sample collection. Moreover, the antibody detection assays can vary considerably between different laboratories in terms of their performance [53].

### 6.3. Polymerase Chain Reaction (PCR)

When infection has been treated with antibiotics, *Campylobacter* may not be detected by culture, but sufficient bacterial DNA may remain in stool, so for this PCR technique is successfully used for the detection. PCR has earlier been used to detect *Campylobacter* species in stool from patients with gastroenteritis [51, 56] but very few studies are available where this method has been applied in patients with GBS [4, 35]. In a recent study, real-time PCR was used to detect *C. jejuni* in fecal samples from a French cohort of patients with GBS [57]. A multiplex PCR assay suitable for mass screening to detect *Campylobacter* directly from chicken feces has been developed [58]. Although PCR is a highly specific and sensitive method, its sensitivity varies among the laboratories and PCR alone cannot exclude the diagnosis of infection [59]. Recently, we tried to detect the association of *C. jejuni *in GBS patients by PCR (19.0%–22.5%), but its sensitivity was found to be low [4, 35]. 

### 6.4. Lymphocyte Transformation Test (LTT)

LTT had earlier been used as a diagnostic modality in many autoimmune and allergic diseases [60–63]. Convalescent excretion of the *C. jejuni *lasts for about 16 days [64] after the onset of diarrhea and the GBS associated with *C. jejuni *typically occurs usually 3-4 weeks after onset of diarrhea [65]. When GBS sets, in most of the cases, *C. jejuni *infection may have cleared but the immune response generated following infection continues during the course of neurological illness. T-cell proliferation when stimulated with *C. jejuni *outer membrane proteins (OMPs) suggests the involvement of T cells in the pathogenesis of the disease. The above-mentioned drawbacks are associated with culture, serology, and PCR in determining antecedent *C. jejuni *infection in GBS patients; therefore, we recently employed LTT together with culture and PCR to assess the efficacy of LTT in diagnosing preceding *C. jejuni* infection in GBS patients after stimulation of lymphocytes with *C. jejuni* OMPs. Our GBS patients had SI values above the cutoff with sensitivity and specificity of the test 77.5% and 95.9%, respectively, [35] under receiver operating characteristic curve. 

**Figure 2:**
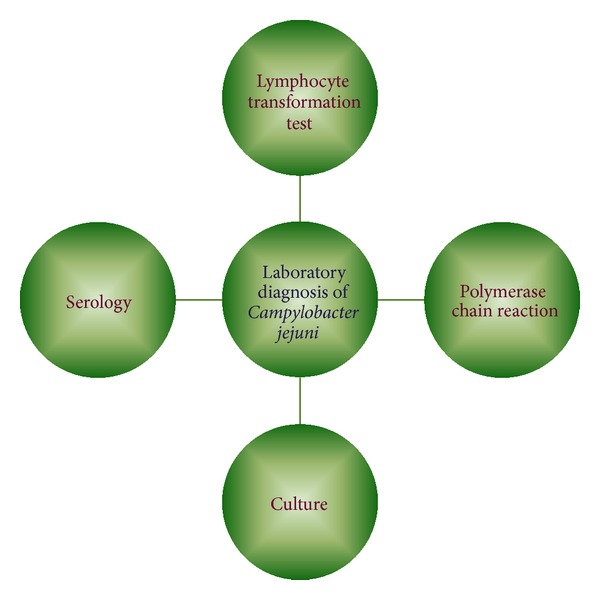
Different laboratory methods for the detection of *Campylobacter jejuni* in patients with Guillain-Barré syndrome.

Different types of established laboratory methods for the detection of *C. jejuni *in GBS patients are summarized in Figure 2.

## 7. Molecular Mimicry between Host Gangliosides and *C. jejuni*

Molecular mimicry is a dual recognition, by a single B- or T-cell receptor, of a microbial component and an antigen of the host, and is the mechanism by which infections trigger cross-reactive antibodies or T cells resulting in autoimmune disease [66] and this phenomenon is proven in GBS [67]. The current hypothesis is that a susceptible human host generates autoantibodies that target both the bacterial ganglioside-like lipooligosaccharide (LOS) structures and human peripheral nerve gangliosides, which triggers axonal degeneration and demyelination of the peripheral nerves. The paralysis or muscle weakness may occur because the immune system breaks the protective Schwann cells surrounding the nerves, allowing enzymes to begin breaking down the myelin “insulation” of nerve axons that help ensure reception and speed of nerve impulses [36]. The pathogenesis of *C. jejuni*-associated GBS has been linked to these antiganglioside autoantibodies produced by ganglioside-like oligosaccharides particularly associated with the Penner (PEN) 19 strain [68]. Earlier, the *C. jejuni *sialyltransferase (Cst-II) was linked to GBS and demonstrated to be involved in the biosynthesis of the ganglioside-like LOS structures. The gene encoding the *C. jejuni *Cst-II, which is required for the generation of ganglioside-like LOS structures GM1 and GD1, is currently the only bacterial marker that has been correlated with GBS [69]. Antiganglioside antibodies were first found in 5/26 (19%) patients with GBS in a study conducted by Ilyas et al. [70]. The wide range of gangliosides to which antibodies have been reported in GBS patients include GM1, AsialoGM1, GM1b, GD1a, GD1b, GD3, GT1a, GT1b, GQ1b, LM1, GalC, and sulfated glucuronyl paragloboside (SGPG). The type of ganglioside mimicry in *C. jejuni *seems to determine the specificity of the antiganglioside antibodies and the associated variant of GBS. *C. jejuni *isolated from patients with pure motor or axonal GBS frequently expresses a GM1-like and GD1a-like LOS [48] that mimics the carbohydrates of gangliosides. Several concepts exist on this issue: (1) anti-GM1 antibodies are irrelevant to the development of GBS and merely exist in patient's serum as secondary events. They are either linked to the disease through preceding infection or as a result of secondary immune response to nerve injury but are independent of its pathogenesis [71]. The most common antibodies are GM1 antibodies; (2) cross-reactivity between antiganglioside antibodies may exist and this have not been fully elucidated. For example, some anti-GM1 may be monospecific whereas others may cross react with other gangliosides; (3), related gangliosides epitopes may exist in both myelin and axolemma membranes in varying concentrations and configurations that can lead to preferential binding of antibody under different circumstances in different individuals. Furthermore, this may change during the course of the disease. For instance, at the nodes of Ranvier axolemma, GM1 may be veiled during the early course of the disease but may become exposed for antibody binding due to paranodal demyelination induced by anti-GM1 or other antibody binding to GM1. Thus, an illness as AIDP could then evolve into AMAN or AIDP with secondary axonal damage. Moran et al. [72] also concluded that the IgG LOS-induced anti-GM1 antibodies bound to sites at the nodes of Ranvier in humans. This is important because other studies have concluded that antibodies bound to nodes of Ranvier disrupt Na^+^ and K^+^ channels, interfering with nerve conduction.

## 8. Host Factors 

Although *Campylobacter jejuni *infections are quite common in general population, the risk of developing GBS is quite low: only 1 in 1000 patients who are exposed to *Campylobacter *infection develops GBS [12, 48]. This strongly suggests that host susceptibility plays an important role in the development of GBS after *C. jejuni *infection. Several lines of evidences point out the importance of host factors in the development and pathogenesis of GBS. First, some *C. jejuni* strains having GM1 ganglioside-like epitopes do not develop antiganglioside antibodies. Second, GBS is rarely found in 2 people within the same family, even within the same village. Finally, although *C. jejuni *lipopolysaccharide (LPS) may exhibit mimicry with gangliosides, why do some people develop a particular form of GBS? In a well-controlled study by Rees et al. [73], 83% of *C. jejuni*-positive GBS patients had significantly higher human leukocyte antigens (HLA) DQB1*03, compared to 49% of the *C. jejuni*-negative GBS patients. We also identified high affinity IgG Fc receptors (Fc*γ*R) and HLA class II molecules, especially DRB1*0701 as novel genetic risk factors for the development of GBS in patients with preceding infections [5]. It now appears likely that the soluble substances other than antibodies may result in nerve damage. Of particular interest are the cytokines which are the molecules with signaling function that coordinate the interplay of immunocompetent cells during an immunoinflammatory response [74]. TNF-alpha −308 G>A and −857 C>T polymorphisms with increased TNF-alpha level may also predict susceptibility to axonal subtypes of GBS [75]. Another study from our centre has also suggested that TLR4 (Asp299Gly) polymorphism increased susceptibility to GBS and AMAN subtype (Thr399Ile) [17]. Several host factors such as matrix metalloproteinase- (MMP-) 2 and MMP-9 and pro- and anti-inflammatory cytokines are the other candidate genes involved in the immune response during different phases of the GBS cases with *C. jejuni* infection [6, 76]. However, geographic variations and different immunogenetic backgrounds may account for different clinical outcomes after *C. jejuni *infection in different parts of the world.

## 9. Immune Response in *C. jejuni-*Associated GBS

The following summarized pathogenetic events are proposed during the *C. jejuni* infection leading to GBS that involve humoral and cellular arms of the immune response such as (i) infection with ganglioside-bearing *C. jejuni* strains, (ii) recruitment of T cells by antiganglioside antibodies producing B cells, (iii) activated T cells that produce cytokines which damage the blood-nerve barrier (BNB), (iv) antiganglioside antibodies accumulate at nodes of Ranvier, (v) opsonization of Schwann cells by antiganglioside antibodies, (vi) invasion of myelin sheath followed by complement-mediated demyelination, and (vii) disruption of Na^+^ and K^+^ channels causing conduction block. Cellular and humoral immune response generated during the pathogenesis of *C. jejuni*-associated GBS is discussed in details.

### 9.1. Cellular Immune Response

GBS is an immune-mediated inflammatory disease affecting the myelin and axons of peripheral nerves. It is generally observed that exogenous antigens may trigger an autoimmune peripheral demyelination by a molecular mimicry-induced loss of tolerance. *C. jejuni *is the most common microorganism implicated in the development of GBS [3, 17, 35]. The host immune response against *C. jejuni *has been assumed to be responsible for the pathogenesis of GBS [77] by inducing cross-reactive antibodies against host gangliosides, and as a result, a cascade of immune-mediated inflammatory responses can be generated by specific immune recognition involving T-lymphocytes, monocytes, and various cytokines responsible for causing demyelination in the host PNS. These cytokines may assist in the disruption of the BNB by which immune cells can infiltrate across the barrier and obtain direct access to the myelin and Schwann cells, thus affecting the peripheral nerve conduction. Anatomically, the BNB is deficient in the distal nerve terminals and nerve roots, and these regions are preferentially affected by an immune attack. Furthermore, Schwann cells can potentially modulate multiple aspects of inflammatory cascade [14] by producing cytokines and toxic substances [78]. Recently, we found significantly higher concentrations of proinflammatory cytokines like IFN-*γ*, IL-1*β*, TNF-*α*, and IL-6 during the progressive phase of the disease [6]. The cytokines produced by the penetrating immune cells in the PNS attack on gangliosides, neurons, or axons lead to severe neurophysiologic abnormalities and the immune-mediated demyelination and axonal damage during GBS [78]. Recent studies have revealed that, during the plateau or recovery period of late stages of GBS, there are a shift from Th1 to Th2 immune response suggesting that Th2-mediated immune response might ameliorate the disease course [6, 79, 80]. The resolution of physiological nerve conduction failure at the nodes of Ranvier leads to rapid recovery in some patients; however, axonal degeneration is associated with slow and incomplete recovery. Recently, the role of Th17 cells, another subset of T helper cells, has been shown and correlated with pathogenesis of the GBS; however, none of the studies reported the presence of these cells in *C. jejuni*-associated GBS. IL17, a signature cytokine produced by Th17 cells, may have synergistic effects with proinflammatory cytokines such as TNF-*α*, IFN-*γ*, and IL-1*β*. IL-17 was found in the sciatic nerves of the experimental autoimmune neuritis (EAN), and the accumulation of IL-17 was correlated with the severity of neurological signs [81], which suggested a pathological contribution of IL-17 to the development of EAN. The frequency of Th17 cells in cerebrospinal fluid (CSF) and the level of IL-17 in plasma were detected significantly higher in active chronic inflammatory demyelinating polyradiculoneuropathy (CIDP) [82] and furthermore, the levels of IL-17 and IL-22 in CSF were correlated with GBS severity [83]. Liang et al. [84] suggested that the TIM-3 pathway influences IL-17 release and Th17 and Th1 differentiation and their cytokine expressions during the pathogenesis of GBS.

Till date, most insights into the immunobiology of inflammatory demyelinating neuropathies have been gained from experimental animal studies. The most frequently employed animal model for GBS is EAN generated in Lewis rats with peripheral myelin or with the purified myelin proteins P0, P2, and PMP22 that proved the role of T lymphocytes in initiating nerve damage. Predominantly, demyelination occurs with low cell doses while the addition of higher cell numbers produces axonal damage and marked endoneurial edema [85–88]. Earlier studies indicated the presence of actively proliferating lymphocytes in blood based on results of the ^3^H-thymidine incorporation assay [85, 89, 90]. In recent times, LTT has been evaluated for the detection of a response to *C. jejuni *antigens in the lymphocytes from GBS patients from our center [35]. Furthermore, we investigated the cytokine profile expressed by the lymphocytes of GBS patients, following stimulation by *C. jejuni *OMPs and compared results with those from progressive and recovery phases of the disease [6]. 

In 1996, Li et al. [39] tried to develop chicken model for GBS; however, the study failed to correlate the pathology with immune response developed during the disease course. To illustrate these initial findings in details, we fed a group of chickens with *C. jejuni *strain isolated from a GBS patient to understand the immunopathogenesis of the disease. In the progressive phase of the disease, we observed that the induction of proinflammatory cytokines (IFN-*γ*, TNF-*α*, and IL-6) in the sciatic nerve of experimental chickens coincided with the accumulation of inflammatory cells such as lymphocytes, macrophages, and neutrophils as well as extensive levels of axonal degeneration and demyelination. Late or recovery phase of the disease was followed by the increased levels of anti-inflammatory cytokines and resolution of inflammation and pathology in the sciatic nerve of the chickens [22]. The observation suggested that these cytokines contribute to recovery of the PNS from damage. Apart from T cells, studies in EAN and in chickens also established the decisive role of macrophages in immune-mediated nerve damage, which are essential in the effector phase of the disease [22, 39, 85, 91, 92]. Macrophages feature prominently in the nerve lesion of GBS. Mechanisms that are operative include phagocytosis and the release of proinflammatory cytokines such as IL-6, TNF-*α*, and IL-1 and other highly active mediators [93–95]. Macrophages are pivotal in initiating the repair phase once the acute inflammatory response has subsided since they clear myelin debris of the nerves and release mitogenic stimuli causing Schwann cell proliferation [96–98].

### 9.2. Humoral Immune Response

Since the first report on antiganglioside antibodies in GBS [70], it had been identified in large group of patients and its association had been established with different clinical subtypes of GBS. In about half of patients with GBS, serum antibodies to various gangliosides have been found in human peripheral nerves. Antibodies specific to peripheral myelin antigens are believed to play a central role in pathogenesis of the disease. Based on the evidence of molecular mimicry between *C. jejuni *LOS and host gangliosides, it has been postulated that autoantibodies induced by the infectious pathogen via shared epitopes are involved in the pathogenesis of GBS. Although the link with antecedent *C. jejuni *infection has remained true for all GBS types, several studies have demonstrated that patients infected with *C. jejuni *more likely develop an axonal subtype than demyelinating subtype of GBS [23, 24]. In addition, antibodies against gangliosides have been found to be associated with *C. jejuni *infection preceding GBS. Accordingly, the response initiated by *C. jejuni *seems particularly related to an antibody-mediated attack targeting neuronal axons in the axonal types of GBS. Promising evidence which supports the concept of specific autoimmune reactions triggered by *C. jejuni *has been obtained from studies based on GBS-linked *C. jejuni *serotypes particularly on *C. jejuni *surface antigenic structures. An alternative hypothesis proposes the importance of humoral immunity in AIDP, especially in the early stages of the disease, whereby antibodies bind to epitopes on the outer surface of Schwann cells inducing complement activation and subsequent myelin destruction prior to macrophage invasion [99]. Antecedent infections, particularly infections with *C. jejuni*, are associated with production of IgG antibodies against gangliosides, especially GM1. Anti-GM1 antibodies, found in approximately 25% of *C. jejuni*-infected GBS patients [24], affect the function of voltage-gated Na^+^ channel at the nodes of Ranvier, thereby resulting in conduction failure [100–102]. It is quite possible that T cells cooperate by opening the BNB to allow circulating autoantibodies access to myelin antigens leading to nerve damage [103] along with nonspecific demyelination by cytokines, activated complement, and other inflammatory mediators generated by a type of acute phase response to *C. jejuni* that further generate cellular immune response against the disease. 

**Figure 3:**
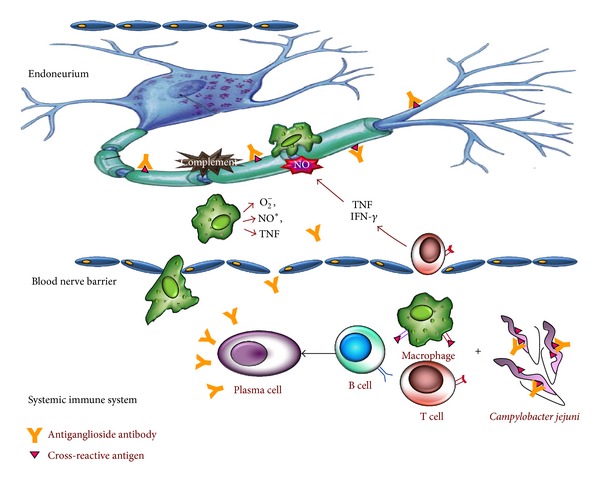
Origin and contribution of antiganglioside antibodies and *C. jejuni* infection to Guillain-Barré syndrome pathogenesis. A bacterial cross-reactive antigen recognized by macrophages and T cells that help B cells to produce antiganglioside antibodies, which penetrate blood-nerve barrier and activate complement. These antibodies bind with specific nerve gangliosides and *C. jejuni* antigen as well. Activated endoneurial macrophages release cytokine and free radicals (nitric oxide), invade compact myelin, periaxonal space, and sometimes block nerve conduction or cause axonal degeneration. Activated T cells release proinflammatory cytokines, fix complement, damage Schwann cell, and ultimately produce dissolution of myelin.

In short, infections with *C. jejuni *may induce an immune response that finally leads to GBS. The immune response depends on certain bacterial factors, such as the specificity of LPS/LOS and patient-related/host factors. Both humoral and cellular immune response associated with autoantibodies and activated lymphocytes, respectively, work in coordination in the pathogenesis of *C. jejuni*-associated GBS. Antibodies to LPS can cross-react with specific nerve gangliosides and can activate complement system. The extent of nerve damage depends on several factors which leads to weakness and may cause conduction disturbances. Upon recovery, the chance of walking unaided after few months can be calculated on the basis of the age of the patient, the presence of diarrhea, and severity of weakness in the first weeks (Figure 3). 

## 10. Animal Models for the *C. jejuni*-Associated GBS

The lack of a good small-animal model that mimics GBS caused by *C. jejuni *in humans has clearly limited our understanding of *C. jejuni *pathogenicity and the host response to infection. Experimental autoimmune neuritis (EAN) is the only available animal model of GBS, in which immune response during various phases of disease has been documented [104, 105]. Though it resembles AIDP histopathologically, there are several disadvantages and dissimilarities to the human disease [106]. Nobody has shown conclusive evidence that autoreactive T-cell response is observed in patients with GBS, indicating that EAN is not a true model of AIDP [67]. Therefore, this model cannot mimic *C. jejuni*-induced GBS. Moreover, due to the scarcity of the nerve biopsy from GBS patients, the mechanism of disease development and its progression after *C. jejuni *infection are least understood. Ferrets colonized with pathogenic *C. jejuni *isolates can exhibit symptoms of disease that are seen in humans, including diarrhea and inflammation [107], but the high cost and lack of suitable reagents and knockout technology to study the host factors involved in the disease diminish the attractiveness of this model. Rabbits have also been reported to develop a sensory neuropathy following immunization with GD1a and GM1 and the findings correspond well with pathological findings for human AMAN; however, this model fails to show demyelination with respect to AIDP [67].

Chickens are the natural reservoirs of *C. jejuni*. In some studies, chicken when used as animal model developed AMAN and AIDP subtypes of the disease. Like AIDP and EAN, inflammatory demyelinating polyradiculoneuropathy in avian is characterized by infiltration of nerve roots and peripheral nerves with macrophages and lymphocytes and, most importantly, a cell-mediated demyelination [108, 109]. Li et al. [39] reported spontaneous paralysis of chickens in farms of GBS patients and subsequently developed a GBS-like paralytic neuropathy in chickens infected with human isolate of *C. jejuni*. Another recent study used chicken as an animal model for GBS and suggested that natural colonization with a GBS-associated *C. jejuni *strain is able to induce specific cross-reactive anti-LOS/ganglioside antibodies in chickens [110]. Furthermore, Bader et al. [108] reported that the paretic phase of avian inflammatory demyelinating polyradiculoneuritis resembles the late-acute phase of human AIDP and is characterized by severe demyelination of peripheral nerves associated with multifocal endoneurial infiltration of lymphocytes and macrophages. Recently, we have also reported that GBS-like neuropathy resembling both axonal and AIDP variants of pathological spectrum can be developed in chickens following *C. jejuni* infection suggesting that chickens may be useful as an experimental animal model to study the immunopathogenesis of *C. jejuni*-associated GBS. We have further showed that enhanced Th1 immune response in early phase of infection contributes to the immune-mediated nerve tissue damage and Th2 immune response in the late phase helps in the repair of the damaged nerve and recovery from *C. jejuni*-associated GBS [22]. Thus, this model is promising to study both bacterial and host genetics and to uncover the pathogenic mechanisms of *C. jejuni*-associated GBS.

## 11. Prevention and Treatment

When outbreak due to *C. jejuni *infections occurs, efforts can be directed towards educating the community about proper food handling techniques and avoiding the consumption of raw milk and/or undercooked poultry. Almost all persons infected with *C. jejuni* recover without any specific treatment. Patients should drink extra fluids as long as the diarrhea lasts. While most *C. jejuni *infections are self-limiting, occasionally a more invasive illness can occur that requires effective antimicrobial therapy. In those cases, antibiotics such as azithromycin or erythromycin and fluoroquinolones can shorten the duration of symptoms if given early in the illness.

Treatment of GBS is required for managing severely paralysed patients who need intensive care and ventilator support and to minimize the nerve damage. Treatments such as plasma exchange and intravenous immunoglobulin (IVIg) are indicated for patients who are unable to walk independently while corticosteroids are largely ineffective in GBS [111]. From the last two decades, plasmapheresis is used as a gold standard treatment for GBS that effectively removes certain inflammatory molecules (cytokines, complement, antibodies, etc.) from the blood [112]. Plasma exchange improves the health leading to reduced ventilated support; moreover, it is a cost- and time-effective treatment [2] for the patient if plasma is exchanged up to four to six volumes in early phase of the disease [3]. In contrast, relapses (25%) are also observed in some patients, which are supposed due to rising antimyelin antibodies in the peripheral blood [113]. Similarly, IVIg in quantity of 0.4 g/kg/day for 5 days continuously is also shown as an effective treatment for the disease [2, 3]. IVIg functions as a suppressor of the immune response in several ways as it interferes with the T lymphocytes proliferation, declines the autoantibody level, suppresses natural killer cell function and antibody-mediated cellular toxicity, and so forth [114–116]; however, complete mechanism of the IVIg is still elusive. Our recent study also supports earlier published data which suggested that IVIgs used for the treatment of GBS suppresse the levels of proinflammatory cytokines such as TNF-*α* and IL-1*β* during recovery which remained relatively high in untreated patients [6]. Several studies suggested superiority of IVIg over plasmapheresis; however, limitations such as tachycardia, headache, and back pain meningeal reactions are associated with this treatment [117].

## 12. Vaccination: A Possible Cause of GBS

In addition to antecedent infections, epidemiological studies have reported development of GBS following vaccinations against several pathogens [2, 99]. Such vaccines include rabies, oral polio, influenza, measles, measles/mumps/rubella, tetanus toxoid and hepatitis B, and other vaccinations. The symptoms of GBS typically start within one day to several weeks following vaccination but usually peak around 2 weeks after the shot is given. Vaccine-induced GBS was first observed within 6–8 weeks of receiving the “swine flu” vaccine during influenza vaccination program in 1976-1977 [118–120]. Further subsequent studies suggested that “GBS is more strongly associated with vaccination for influenza” than for any other vaccine, but the exact reason for this association remains unknown. Analysis of vaccine program during 1993-1994 in USA accounted a slightly increased risk of GBS within the 6 weeks after immunization [121]. A more recent study also showed that influenza A (H1N1) 2009 monovalent inactivated vaccines were associated with a small increased risk of GBS. This finding translated to about 1.6 excess cases of GBS per 100,000 vaccinated candidates [122]; however, conflicting reports do exist. Studies of influenza vaccines used in subsequent years, however, have found small or no increased risk of GBS [123, 124]. In 2011, the Institute of Medicine reviewed data through the 2008-2009 influenza seasons and concluded that “the evidence is inadequate to accept or reject a causal relationship between influenza vaccine and GBS” [125]. In contrast, the evidence favored an association between oral polio vaccine and tetanus toxoid-containing vaccines and GBS. However, recent evidence from large epidemiological studies and mass immunization campaigns in different countries found no correlation between oral polio vaccine or tetanus toxoid-containing vaccines and GBS [123]. Hepatitis vaccine and the meningococcal conjugate vaccine also carry a risk of GBS [3, 126]. Furthermore, rabies vaccine prepared from the infected brain tissues of adult animals had an increased risk of inducing GBS due to the contamination with myelin antigens [127] but newer formulations of rabies vaccine, derived from chick embryo cells, do not appear to be associated with GBS at greater than the expected rate [123]. Comparisons with expected rates of GBS, however, were inconclusive for an increased risk, and lack of controlled epidemiological studies makes it difficult to draw conclusions about a causal association. Existing data for other vaccines are available based on isolated case reports or small groups related to immunizations, and no conclusion about causality can be drawn.

## 13. Conclusions and Future Directions

*C. jejuni* infection is the predominant antecedent infection in GBS. It has been identified in 30%–50% of GBS patients and supposed as a potential predictor of poor outcome. Also about 20% of GBS patients are left with a functional disability and 60% report severe fatigue at 12 months. A more severe autoimmune response and greater axonal damage are mostly observed in *C. jejuni*-associated GBS. This is problematic in poorer countries where patients may have limited access to healthcare and treatment required for GBS. Therefore, the appropriate procedures must be developed to reduce the incidence of *C. jejuni-*associated GBS. The transmission of *C. jejuni* may be prevented by improving sanitation, well-cooked poultry products, disinfection of water, and public health warnings about hazards of raw milk consumption. The pathogenesis of the disease is believed to involve molecular mimicry between epitopes on *C. jejuni *LPS and neural gangliosides, resulting in immunologic damage to the peripheral nerve. Antibody- and cell-mediated immune responses are believed to produce degeneration of the nerve and interruption of neurotransmission. Studies should be made to investigate the emerging role of Th17 cells in reference to axonal and demyelinating subtypes, as well as infections particularly with *C. jejuni*. The infection is often associated with the presence of antibodies against GM1, which may target and injure the peripheral nerves resulting in the severity of the disease. Possibly to prevent *C. jejuni*-induced GBS, efforts should be directed towards markedly reducing the numbers of severely disabled survivors of GBS. Analysis of the expression of *C. jejuni* genes involved in LOS biosynthesis should be helpful in designing drugs useful in treating these conditions. For continued advancement in this field, researchers will need to work in a collaborative effort to dissect the mechanisms of molecular mimicry and immune-mediated nerve damage.
